# 融合液相色谱-串联质谱技术与室间质量评价的临床检验专业本科毕业设计教学实践

**DOI:** 10.3724/SP.J.1123.2025.06030

**Published:** 2026-03-08

**Authors:** Jie SHI, Mo WANG, Qiaozhen XUE, Shunli ZHANG, Yanwei HU

**Affiliations:** 1.首都医科大学附属北京朝阳医院，北京 100020; 1. Beijing Chao-Yang Hospital，Capital Medical University，Beijing 100020，China; 2.北京市临床检验中心，北京 100020; 2. Beijing Center for Clinical Laboratory，Beijing 100020，China

**Keywords:** 液相色谱-串联质谱, 25-羟基维生素D_2_, 25-羟基维生素D_3_, 室间质量评价, 临床检验, 教学创新, liquid chromatography-tandem mass spectrometry（LC-MS/MS）, 25-hydroxyvitamin D_2 _（25（OH）D_2_）, 25-hydroxyvitamin D_3 _（25（OH）D_3_）, external quality assessment （EQA）, clinical laboratory science, teaching innovation

## Abstract

本研究旨在探索液相色谱-串联质谱（LC-MS/MS）技术在检验专业本科生毕业设计教学中的应用与教学创新。以25-羟基维生素D_2_（25（OH）D_2_）和25-羟基维生素D_3_（25（OH）D_3_）室间质量评价（EQA）样本的研制为教学载体，构建了以科研任务和临床工作为驱动的实验教学模式。教学设计涵盖样品制备、仪器操作、数据处理与结果评价等完整环节，使学生在实践中掌握LC-MS/MS检测的核心技术流程与质量控制方法。通过实验项目的实施，学生参与了EQA样本的制备、参考方法定值、均匀性与稳定性评价及EQA成绩的分析，全面体验了从实验设计到结果评价的过程。研究结果表明，所制备EQA样本具有良好的均匀性与稳定性，满足中国合格评定国家认可委员会（CNAS）质量标准要求。在“2024年北京市血清维生素D检测”EQA活动中，北京地区共有16家医疗机构实验室参加。教学实践显示，该项目有效提升了学生在实验设计、仪器操作、数据分析及质量评价等方面的综合能力，增强了科研思维与团队协作意识。基于“以研促教、产教融合”的教学理念，将高端分析技术引入本科教学体系，不仅优化了实验课程内容与教学环节，也拓展了学生在临床检验与科研工作中的应用视野。本研究为检验专业本科生的实践教学提供了可借鉴的教学范式，对推动先进分析技术在医学检验教育中的推广与应用具有重要参考价值。

## Abstract (en)

This study aimed to explore the application and pedagogical innovation of liquid chromatography-tandem mass spectrometry （LC-MS/MS） in undergraduate thesis training for medical laboratory science students. Using the development of 25-hydroxyvitamin D_2_ （25（OH）D_2_） and 25-hydroxyvitamin D_3_ （25（OH）D_3_） external quality assessment （EQA） samples as a teaching platform， an experimental teaching model driven by research tasks and clinical practice was established. The teaching design encompassed the entire workflow， including sample preparation， instrument operation， data processing， and result evaluation， enabling students to acquire hands-on experience with core LC-MS/MS techniques and quality control procedures. Through the implementation of the experimental project， students participated in EQA sample preparation， reference method-based quantification， homogeneity and stability assessments， and EQA result analysis， thereby gaining comprehensive exposure to the process from experimental design to result interpretation. The results demonstrated that the prepared EQA samples exhibited satisfactory homogeneity and stability， meeting the quality standards established by the China National Accreditation Service for Conformity Assessment （CNAS）. In the “2024 Beijing Serum Vitamin D EQA” activity， a total of 16 medical laboratory institutions in Beijing participated. Teaching practice revealed that this project significantly enhanced students’ comprehensive abilities in experimental design， instrument operation， data analysis， and quality assessment， while also fostering scientific thinking and teamwork. By introducing advanced analytical techniques into the undergraduate curriculum under the educational concept of “research-driven teaching and integration of industry and education”， this approach not only optimized course content and experimental procedures but also broadened students’ practical perspectives in clinical laboratory and research applications. This study provides a replicable teaching model for undergraduate laboratory education and offers valuable insights for promoting the integration of advanced analytical technologies into medical laboratory science training.

在现代医学持续发展的背景下，临床检验诊断学作为医学体系的重要组成部分，在疾病的准确诊断、治疗方案的制定及疗效与预后评估中发挥着不可替代的作用^［1，2］^。随着生命科学研究的深入和精准医学理念的推进，临床检验对检测技术的灵敏度、特异性和可重复性提出了更高要求^［3］^。液相色谱-串联质谱（liquid chromatography-tandem mass spectrometry，LC-MS/MS）作为代表性先进分析技术，依托色谱分离与质谱检测的优势，能够在复杂生物样本中实现多组分的精确定量与定性，已成为临床检验与科研分析的重要支撑手段^［4］^。

通过在教学中引入先进仪器分析技术，学生能够在实践中理解色谱分离与质谱检测原理，熟悉实验数据的处理与质量控制流程，提升其解决复杂问题的综合能力^［5］^。25-羟基维生素D（25-hydroxyvitamin D，25（OH）D）是评价人体维生素D营养状况的核心指标，其检测结果直接影响骨代谢疾病、免疫相关疾病及慢性病的临床管理^［6-9］^。其中，25-羟基维生素D_2_（25（OH）D_2_）和25-羟基维生素D_3_（25（OH）D_3_）是维生素D在血清中的主要储存和代谢形式。LC-MS/MS方法能够特异性区分这两种结构类似的化合物，避免免疫分析法的交叉反应干扰，其定量结果可通过国际标准参考物质实现量值溯源，因此被广泛认可为测定25（OH）D的“金标准”方法^［10］^。然而，目前临床实验室仍以化学发光法为主，其检测结果在不同实验室间存在较大差异（变异系数可达15%~20%），对结果可比性造成影响。

随着医学检验学科的不断拓展和检测技术的迭代更新，检验专业人才培养在创新意识与技术能力方面面临新的挑战^［11，12］^。检验专业本科生作为未来医学检验体系的骨干力量，亟须在校阶段掌握包括LC-MS/MS在内的前沿检测技术，以加深其对临床检验质量控制和结果可靠性的理解，同时提升实验设计、数据分析及科研创新能力，从而为其未来在医院检验科、第三方检测机构及科研岗位的职业发展奠定坚实基础^［13］^。

基于此，本研究将LC-MS/MS技术引入医学检验专业本科生毕业设计教学，以25（OH）D_2_和25（OH）D_3_室间质量评价（external quality assessment， EQA）样本研制为实践载体，探索科研与教学深度融合的创新教学模式^［12，14］^，同时提升实验设计、数据分析及科研创新能力，为高水平检验医学人才培养提供新的教学范式^［11，13］^。

## 1 实验部分

### 1.1 仪器、试剂与材料

RZ-500超高效液相色谱-质谱联用系统（上海睿康生物科技有限公司），用于常规LC-MS/MS检测；AB5500液相色谱-质谱联用系统，包括Nexera-XR液相色谱系统（岛津公司，日本）与5500 Q-Trap三重四极杆质谱仪（AB Sciex公司，美国），用于参考方法定值。BCE55I-10CN天平（赛多利斯公司，德国），H17.5R高速离心机（上海卢湘仪公司），MS5磁力搅拌器（群安仪器），AN25D020-96孔氮吹仪（纳鸥科技），EX-48全自动核酸提取仪、脂溶性维生素样本释放剂（包装规格FSV_MB2 96人份/盒，包括稳定剂、复溶液Ⅰ、96孔预分装提取试剂、沉淀剂、复溶液Ⅱ、搅拌套、96孔深孔板、96孔V型板、热封铝箔膜和封口胶，北京华大吉比爱生物技术有限公司）。

25（OH）D_2_标准品（纯度≥95%）和25（OH）D_3_标准品（纯度≥98%）购自美国Sigma-Aldrich公司；同位素内标［6，19，19-D_3_］-25（OH）D_2_（纯度≥97%）和［6，19，19-D_3_］-25（OH）D_3_（纯度≥97%）购自美国Cambridge Isotope Laboratories， Inc公司；甲醇（HPLC级）、乙腈（HPLC级）、乙醇（HPLC级）、甲酸（纯度≥99%）和正己烷（HPLC级）购自美国Fisher Scientific公司。碳酸钠（纯度≥95%）和七水合硫酸锌（纯度≥98%）购自北京益利精细化学品有限公司。

### 1.2 室间质量评价

EQA是实验室质量管理体系的重要组成部分，其基本原理是通过标准化样本比对，识别实验室间的系统误差来源，并通过数据反馈机制促进检测方法与质量体系的改进^［14，15］^。

EQA的实施通常包括4个关键步骤：（1）样本设计与分发：制备与临床样本基质相似、具有良好均匀性和稳定性的样本，并分发至各参加实验室；（2）检测与数据回报：实验室使用常规检测方法（如免疫法、LC-MS/MS法）对样本进行检测，并按要求上报检测结果；（3）数据统计与评价：采用稳健统计学方法（如中位数、标准化四分位距）剔除异常值，对各实验室检测值与靶值之间的偏差进行分析，并依据预设的可接受范围（±25%，国家卫生健康委临床检验中心给出的维生素D的EQA评价标准）判断检测结果是否合格；（4）结果反馈与改进：EQA组织机构根据统计结果向实验室反馈偏差信息，实验室据此优化检测方法、调整校准体系或改进操作流程，实现质量持续改进。

在25（OH）D_2_和25（OH）D_3_的检测中，EQA能够有效反映不同检测方法之间的系统性差异。参考方法赋值（如LC-MS/MS）可用于确定靶值，并以此校准化学发光法等常规检测方法，确保检测结果的可比性和可溯源性。通过建立统一的EQA体系，可促进维生素D检测的标准化与结果一致性，从而提升临床诊断的可靠性和实验室间结果的可追溯性。

### 1.3 样本制备

称取25（OH）D_2_和25（OH）D_3_各1 mg，分别溶解于1 mL甲醇中，制备1 mg/mL储备液。随后将储备液稀释1 000倍，得到终质量浓度1.00 μg/mL的标准工作液（即25(OH)D_2_ 2.427 μmol/L，25(OH)D_3_ 2.496 μmol/L）。

收集首都医科大学附属北京朝阳医院检验科检测后血清共300 mL，检测其本底水平后平均分为3份（各100 mL），其中一份在磁力搅拌器上加入25（OH）D_2_标准溶液0.65 mL，编号为202411；一份在磁力搅拌器上加入25（OH）D_3_标准溶液1.95 mL，编号为202412；另一份不添加标准品，作为低浓度样品，编号为202413。各组样品均在室温下搅拌30 min后静置，每组分装150支，每支0.65 mL，备用。本研究经首都医科大学附属北京朝阳医院伦理委员会批准，审批号（2023-科-410）。

### 1.4 常规LC-MS/MS方法检测样本均匀性和稳定性

#### 1.4.1 样本处理

取出96孔预分装提取试剂，3 000 r/min离心1 min，使试剂及磁珠均集中到孔板底部。取300 μL稳定剂工作液和300 μL待测样本分别加入96孔预分装提取试剂的第3、9列孔中，使全自动核酸提取仪运行如下提取程序：混匀，活化，平衡，提取，3次淋洗，洗脱。仪器程序结束后，将第6、12列孔中的洗脱液吸取到96孔深孔板中，置于96孔氮吹仪上吹干。加入80 μL复溶液Ⅱ的稀释液（复溶液Ⅱ与水的体积比为8∶2），封口胶封板，涡旋振荡10 min，离心（4 000 r/min，4 ℃）10 min，然后将全部溶液转移至96孔V型板，封口膜封板，用RZ-500液相色谱-质谱联用仪检测。

#### 1.4.2 色谱条件

Boltimate岛津C18色谱柱（50 mm×2.1 mm，2.7 μm）；流动相：A相为0.1%甲酸水溶液，B相为0.1%甲酸甲醇；梯度洗脱程序：0～0.2 min，80%B；0.2～2.5 min，80%B～100%B；2.5～5.0 min，100%B；5.0～5.1 min，100%B～80%B；5.1～6.5 min，80%B。流速0.4 mL/min。进样体积为15 μL。

#### 1.4.3 质谱条件

大气压化学电离（atmospheric pressure chemical ionization， APCI）离子源，正离子、多反应监测（multiple reaction monitoring， MRM）模式，离子源鞘气30 Arb，辅助加热气5 Arb，吹扫气1 Arb，离子传输管温度400 ℃，离子源内腔温度400 ℃，25（OH）D_2_和25（OH）D_3_的监测离子对分别为*m/z* 395.2/377.4和*m/z* 383.4/365.4，对应的碰撞能量分别为15.67 V和14 V。

### 1.5 样本均匀性和稳定性评价

按照CNAS-GL003《能力验证样品均匀性和稳定性评价指南》的评价和统计方法对样本进行均匀性和稳定性评价；在方法精密度满足CNAS-CL02-A003《医学实验室质量和能力认可准则在临床化学检验领域的应用说明》要求情况下，按照1.2节EQA评价方法进行样本抽取和检测。

#### 1.5.1 均匀性实验评价

均匀性评价主要利用方差分析，以判断有无瓶间差。考虑到实际工作的预期标准差（standard deviation， SD）的要求，方差分析显示有瓶间差时，如果SD不大于0.3*σ*（*σ*=总允许误差（total error allowable，TEA）×均值），仍可认为瓶间差合格，即均匀性良好。在本项研究中，我们从已经制备好的样本中，抽取202411和202412样本各10支，使用常规LC-MS/MS方法检测血清25（OH）D_2_和25（OH）D_3_，每支检测两次，再使用方差分析进行均匀性验证。

#### 1.5.2 稳定性实验评价

稳定性评价主要监测被测物浓度在运输途中和短期存储条件下是否发生变化，通过选择不同的温度点和不同的时间点对样本进行研究，如果该研究点被测物的检测值与原点检测值相比小于0.3 *σ*，则认为该样本具有良好的稳定性。在本项研究中，选择202411和202412不同浓度水平的EQA样本进行稳定性检验。其中，“0时样本”是从-80 ℃冰箱取出样本恢复室温后立即检测，另取同样两批号样本于“37 ℃下存储2 h”“2~8 ℃下存储4 h”“-20 ℃下存储5个月”，每个点各取3支，各检测2次。

### 1.6 25（OH）D_2_和25（OH）D_3_定值

25（OH）D_2_和25（OH）D_3_ EQA样本采用国家卫生健康委临床检验中心建立的参考方法进行定值^［16］^：首先使用常规LC-MS/MS方法测定每个血清样本中25（OH）D_2_和25（OH）D_3_的浓度，然后使用AB5500液相色谱-质谱联用系统进行参考方法定值：根据其浓度将适量（100~800 μL）的样本精确称重至安瓿瓶中，加入30~50 μL内标溶液（100 μg/mL），以获得预期的天然/标记物比例（对于25（OH）D_2_和25（OH）D_3_，该比例范围为0.25~2.5）。之后加入Na_2_CO_3_水溶液和去离子水至1 mL终体积。室温下平衡1 h。最后采用蛋白质沉淀和液-液萃取从血清中提取目标化合物，使用AB5500液相色谱-质谱联用系统进行LC-MS/MS分析。

### 1.7 EQA样本的分发、检测和结果上报

样本以干冰冷冻快递至各参加EQA活动的实验室。每个实验室收到3个批号（202411、202412、202413）、每批3支样本，共9支。

要求参加实验室在接到样本后，连续3日进行检测，每日测定每个批号各1支，每支重复测定3次，1周内测定完毕，当日未检测样本于-20 ℃存储。测定当日将样本在室温放置30 min，待其完全融化，轻轻上下颠倒混匀，再进行检测。测定方法为各参加实验室检测临床样本的常规方法。

待所有样本测定完成后，在北京市临床检验中心网站（https：//corelab.clinet.com.cn/）登录实验室用户名和密码后，填写电子版回报表，上报结果。

### 1.8 EQA结果分析

采用2003版CLInetEQA软件，对参加实验室回报的数据进行稳健统计分析：首先在软件“网络下载”模块中，将各实验室在网站上回报的结果下载至本地存储器，之后在“质评活动”模块中，填写评价标准为“±25%”，然后在“数据分析和计算”模板中，手工添加靶值（参考方法定值的结果），最后在软件“实验结果”模块中，点击“计算”，软件将自动计算所有参加实验室的EQA成绩。

## 2 结果与讨论

### 2.1 常规LC-MS/MS方法的精密度

选择3个水平的样本，每个样本测定5次，连续测定3批，25（OH）D_2_的标准溶液浓度为5.16、6.88、13.88 nmol/L，批内精密度为5.28%、3.92%、2.95%，总精密度5.40%、5.93%、5.01%。25（OH）D_3_的标准溶液浓度为35.34、43.04、58.66 nmol/L，批内精密度为3.32%、2.56%、4.16%，总精密度为4.55%、5.06%、5.88%。

### 2.2 样本均匀性评价

样本均匀性评价结果见附表S1（www.chrom-China.com）和附表S2，方差分析统计的结果见表1和表2。从结果可以看出，两个批号所涉及25（OH）D_2_和25（OH）D_3_的*F*值（*F=*组间方差/组内方差）均小于*F*界值（*F*_（9，10）_=3.02），说明制备的EQA样本均匀性符合要求。

**表1:** 25（OH）D_2_ EQA样本方差分析结果表

Batch No.	Source of variation	Degree of freedom	Sum of squares	Mean square	*F*
202411	between the samples	9	0.518	0.058	1.78
	within the sample	10	0.323	0.032	
202412	between the samples	9	24.875	2.764	1.90
	within the sample	10	14.475	1.448	

*F*=between-group variance/within-group variance.

**表2:** 25（OH）D_3_ EQA样本方差分析结果表

Batch No.	Source of variation	Degree of freedom	Sum of squares	Mean square	*F*
202411	between the samples	9	5.769	0.641	2.46
	within the sample	10	2.609	0.261	
202412	between the samples	9	113.025	12.558	1.08
	within the sample	10	116.374	11.637	

### 2.3 样本稳定性评价

样本的存储稳定性评价结果见附表S3~S6。结果表明，25（OH）D_2_和25（OH）D_3_在“37 ℃下2 h”“2~8 ℃下4 h”“-20 ℃下5个月”条件下的检测值与原点检测值相比小于0.3*σ*，稳定性良好，说明EQA样本于上述温度和时间的运输和储存能够满足稳定性要求。

### 2.4 样本定值结果及可接受范围

将制备的EQA样本采用参考方法定值^［16］^，每个批号选取3支样品，每支样品重复测量3次，并以所有测定值的平均值作为靶值。根据±25%评价标准，计算可接受的上下限。由表3可以看出，各批次EQA样本的25（OH）D_2_、25（OH）D_3_以及两者相加计算得出的总25（OH）D的靶值呈现明显的浓度梯度，且总25（OH）D的靶值浓度也对应了中华人民共和国卫生行业标准WS/T677-2020《人群维生素D缺乏筛查方法》^［17］^中人群维生素D的不同水平的参考范围（25（OH）D≥50 nmol/L为正常，30～50 nmol/L为不足，<30 nmol/L为缺乏）。

**表3:** 25（OH）D EQA样本靶值及可接受限

Project name	Batch No.	Target value/（nmol/L）	Acceptable lower limit/（nmol/L）	Acceptable upper limit/（nmol/L）
25（OH）D_2_	202411	21.30	15.98	26.63
	202412	10.30	7.73	12.88
	202413	6.42	4.82	8.03
25（OH）D_3_	202411	17.05	12.79	21.31
	202412	63.00	47.25	78.75
	202413	15.11	11.33	18.89
Total 25（OH）D	202411	38.35	28.76	47.94
	202412	73.30	54.98	91.63
	202413	21.53	16.15	26.91

由此可见，本研究不仅成功制备了满足均匀性和稳定性评价要求的EQA样本，同时总25（OH）D项目也覆盖了正常、不足和缺乏这3种不同浓度水平的人群，符合EQA样本对临床样品浓度分布的要求，为实验室间结果比对提供了可靠的参考材料。

### 2.5 EQA成绩

北京市临床检验中心于2024年下半年执行了1次血清25（OH）D_2_和25（OH）D_3_检测的EQA活动计划，参加的实验室及检测结果情况见表4。可以看出，参加实验室总数为16家。由于仅质谱检测方法可测定25（OH）D_2_和25（OH）D_3_的浓度，因此回报25（OH）D_2_或25（OH）D_3_检测结果的实验室仅有2家，回报率12.5%；多数实验室仍采用化学发光等方法检测总25（OH）D，回报总25（OH）D检测结果的实验室有15家，回报率为93.75%。

**表4:** 参加的实验室及检测结果

Laboratory No.	25（OH）D_2_/（nmol/L）			25（OH）D_3_/（nmol/L）			Total 25（OH）D/（nmol/L）			Testing method
	202411	202412	202413	202411	202412	202413	202411	202412	202413	
1	22.81	2.12	2.81	20.29	80.37	21.05	43.10	82.49	23.87	LC-MS/MS
2				17.09	64.76	17.73				LC-MS/MS
3							26.88	71.56	23.49	AECL
4							65.73	198.67	44.17	AECL
5							29.63	69.69	16.09	ECL
6							73.20	175.00	55.51	ECL
7							24.25	69.30	19.10	ECL
8							27.84	83.38	20.28	ECL
9							32.37	71.84	19.72	CLIA
10							29.18	66.79	22.36	ELISA
11							29.02	67.43	22.45	ELISA
12							35.55	78.77	23.46	MCLIA-AMPPD Marked
13							28.59	85.89	18.80	MEIA
14							24.43	76.70	16.75	MEIA
15							64.06	152.56	44.21	CLIA
16							29.11	39.15	20.02	other
Pass rate/%	100.00	0	0	100.00	50.00	50.00	46.67	73.33	73.33	

LC-MS/MS： liquid chromatography-tandem mass spectrometry method； AECL： acridinium ester chemiluminescence method；ECL： electrochemiluminescence method；CLIA： chemiluminescence immunoassay method； ELISA： enzyme-linked immunosorbent assay； MCLIA-AMPPD Marked： micro-particle chemiluminescence-3-（2′-spiroadamantane）-4-methoxy-4-（3″-phosphoryloxy）phenyl-1，2-dioxetane marked； MEIA： micro-particle enzyme immunoassay method.

从总25（OH）D的各实验室结果可见，批号202411的合格率最低，为46.67%；202412的合格率为73.33%；202413的合格率为73.33%。结合参考方法定值结果分析可以看出，202411的25（OH）D_2_浓度最高，化学发光法对高浓度25（OH）D₂的检测存在一定局限性，因此该批次的总25（OH）D的合格率相对较低。

## 3 教学实践与反思

### 3.1 项目背景与教学目标

在毕业设计教学环节中，首先向学生介绍25（OH）D_2_和25（OH）D_3 _项目EQA样本研制的科学与临床背景。维生素D在维持钙磷代谢平衡、骨骼健康及免疫功能中发挥关键作用，其缺乏或过量与佝偻病、骨质疏松及心血管疾病等多种疾病密切相关。准确测定血清中25（OH）D_2_和25（OH）D_3_水平对于疾病的诊断、治疗及预防具有重要意义。EQA是保障临床检验结果准确性和实验室间可比性的核心手段，而高质量的EQA样本制备是其成功实施的基础。本教学项目以25（OH）D_2_和25（OH）D_3_ EQA样本研制为载体，引导学生在实践中理解如何通过统一定值方法、质量评价标准和量值溯源体系实现检测结果的一致性与可比性，并掌握先进检测技术的应用流程。

本课程的教学目标包括：知识目标，掌握LC-MS/MS的基本结构、工作原理及操作流程，理解色谱分离与质谱检测的基本原理及其在临床检验中的应用；技能目标，学习25（OH）D₂和25（OH）D₃ EQA样本的制备、定值及质量评价方法，并能够独立完成样本测定、数据统计与分析；素养目标，熟悉EQA体系在实验室质量控制中的作用与评价原则，培养严谨的实验态度和科研思维。

学生在实验过程中需严格按照标准操作规程完成样品制备、仪器校准及检测流程，确保数据的可重复性与准确性。教学环节不仅关注仪器操作能力的培养，更注重学生对实验流程中质量控制环节的理解与掌握。学生需熟悉EQA样本的发放流程，掌握CLInetEQA软件的使用，了解稳健统计方法的原理，并能熟练应用这些方法对实验室回报结果进行统计分析和评价。

### 3.2 教学设计与实验实施

#### 3.2.1 实验分阶段安排

首月 理论学习与仪器培训：系统学习LC-MS/MS基本原理、仪器结构及操作方法，了解EQA意义及25（OH）D在临床诊断中的价值，结合文献掌握国内外检测标准及研究进展，为后续实验操作奠定理论基础。

次月 样本制备：学生按要求收集血清样本，配制标准工作液，经充分涡旋混匀后分装，-80 ℃冰箱储存备用，确保样本均匀性和长期稳定性。此环节强调样本前处理标准化，避免操作误差对检测结果的影响。

第3~7个月 在不同储存温度和时间条件下设计样本检测实验，进行样本测定、重复实验及初步数据记录；随后统一汇总测定结果，进行统计分析以验证样本的均匀性和稳定性，为EQA靶值设定提供可靠依据。

第8个月 定值实验与EQA结果分析：学生学习参考方法（基于同位素稀释的液相色谱-串联质谱法），对样本进行准确定值测定，参与数据统计与分析，撰写实验报告并总结问题与改进建议，完成全流程实验实践。

#### 3.2.2 关键实验环节设计

标准化操作考核：学生严格遵循操作规范，如移液精度、色谱柱平衡、质谱参数设定等，教师现场评分，确保实验操作可重复、数据可靠。

EQA成绩分析：通过汇总统计参加实验室的回报数据，计算各批样本的偏差、均值和变异范围，并与参考靶值进行比对，理解实验室间数据差异和方法可比性，从而掌握质量控制的基本原理和实验数据分析技能。

#### 3.2.3 安全与协作管理

教学中强调有机溶剂、质谱设备及相关耗材的安全操作，要求学生配备护目镜、防护手套并在通风环境中作业，确保实验安全。通过定期汇报与小组讨论，教师了解学生实验进度与存在问题，促进团队协作与问题解决能力的提升。

### 3.3 教学反思

本实验教学以“科研项目化教学”为核心理念，通过研制25（OH）D_2_和25（OH）D_3_的EQA样本，并将其应用于2024年北京市临床检验中心EQA计划中，实现了将临床实际需求与实验教学相互融合，引导学生在真实科研任务和临床工作中，完成从EQA样本制备源头到结果分析评价的全流程训练，实现了“教学-科研-临床”三位一体的育人目标。学生在任务驱动和问题导向的学习中，不仅掌握了核心实验技能，更逐步形成了基于证据的科研思维和自主解决实际问题的能力。

然而，本次教学实践也反映出若干问题：一方面，由于LC-MS/MS设备资源有限，学生实际操作机会不足，对仪器原理理解不深；另一方面，部分学生理论基础薄弱，对色谱、质谱等方法学原理理解不透彻，同时在数据分析、统计推断等跨学科知识方面存在短板，导致“重操作、轻思考”的现象，影响了科研能力的系统养成。

为解决这些问题，本课程采取了多项结构化改进措施：在EQA样本制备前，要求学生基于文献调研，独立完成标准品工作液的浓度配制等方案设计；在仪器分析方面，除常规检测的软件操作，定期带领学生进行仪器设备的内部维护保养，更换不同色谱柱和流动相，引导学生观察以上操作对结果的影响，理解方法建立背后的物理化学原理；在EQA成绩统计环节，指导学生操作CLInetEQA，设置靶值、进行稳健统计，审视结果的可靠性与偏倚来源；实验结束后，通过访谈收集学生对教学模式的反馈，结果显示大部分学生认为该模式有效促进了实验技能提升和科研能力培养。这些举措共同推动学生从被动执行者向主动研究者转变。

## 4 结语

LC-MS/MS技术作为现代医学检验的重要分析手段，在临床诊断和科研中具有不可替代的作用。本研究以25（OH）D的 EQA样本研制为案例，将LC-MS/MS系统融入本科生毕业设计教学中，通过实验设计、操作实践和数据分析的全流程训练，使学生掌握技术核心原理、操作流程及质控方法，同时培养科研思维和临床问题解决能力。教学实践表明，学生能够在指导下完成从样本制备到数据分析的全流程实验，充分体现教学成果的转化价值。本模式具有一定的推广潜力，可在兄弟院校开展合作教学或科研训练项目，实现经验共享和教学资源互补。

本研究为检验专业本科生实践教学提供了创新模式，同时在有限的实验室条件下，为临床实验室25（OH）D检测的标准化和EQA体系完善提供了技术支持。尽管存在样本代表性和方法普适性等局限（如样本量仅16家实验室，可能影响结果的广泛代表性，时间跨度较短），但这一教学实践为培养适应现代检验医学发展的高素质人才奠定了基础。

随着医学检验学科与精准医疗、组学分析的深度融合，传统实验教学正逐步向创新性、研究型方向转变。本教学模式为医学检验专业本科生提供了一个以真实科研为驱动的实践平台，有助于学生在“做中学、研中悟”中熟悉科研的完整流程并掌握关键技能。未来，将进一步引入代谢组学与多组学联合分析内容，探索基于真实临床数据的科研训练新模式，实现从单一实验技能培养到科研思维塑造的转变，最终为培养具备临床意识、科研能力与创新精神的高水平医学检验人才提供可复制、可推广的教学范式。
